# Non-small cell lung cancer cells survived ionizing radiation treatment display cancer stem cell and epithelial-mesenchymal transition phenotypes

**DOI:** 10.1186/1476-4598-12-94

**Published:** 2013-08-16

**Authors:** Roberto Gomez-Casal, Chitralekha Bhattacharya, Nandita Ganesh, Lisa Bailey, Per Basse, Michael Gibson, Michael Epperly, Vera Levina

**Affiliations:** 1 University of Pittsburgh Cancer Institute, Pittsburgh, PA 15213, USA; 2 Department of Medicine, Pittsburg, PA 15213, USA; 3 Departments of Pathology, University of Pittsburgh, Pittsburgh, PA 15213, USA; 4 University of Pittsburgh Cancer Institute, Hillman Cancer Center, Rm. 1.19c, 5117 Center Ave, Pittsburgh, PA 15213, USA

**Keywords:** Non-small cell lung cancer, Ionizing radiation, Cancer stem cell, Epithelial-mesenchymal transition

## Abstract

Ionizing radiation (IR) is used for patients diagnosed with unresectable non small cell lung cancer (NSCLC), however radiotherapy remains largely palliative due to radioresistance. Cancer stem cells (CSCs), as well as epithelial-mesenchymal transition (EMT), may contribute to drug and radiation resistance mechanisms in solid tumors. Here we investigated the molecular phenotype of A549 and H460 NSCLC cells that survived treatment with IR (5Gy) and are growing as floating tumor spheres and cells that are maintained in a monolayer after irradiation.

Non-irradiated and irradiated cells were collected after one week, seeded onto ultra low attachment plates and propagated as tumor spheres. Bulk NSCLC cells which survived radiation and grew in spheres express cancer stem cell surface and embryonic stem cell markers and are able to self-renew, and generate differentiated progeny. These cells also have a mesenchymal phenotype. Particularly, the radiation survived sphere cells express significantly higher levels of CSC markers (CD24 and CD44), nuclear β-catenin and EMT markers (Snail1, Vimentin, and N-cadherin) than non-irradiated lung tumor sphere cells. Upregulated levels of Oct-4, Sox2 and beta-catenin were detected in H460 cells maintained in a monolayer after irradiation, but not in radiation survived adherent A459 cells.

PDGFR-beta was upregulated in radiation survived sphere cells and in radiation survived adherent cells in both A549 and H460 cell lines. Combining IR treatment with axitinib or dasatinib, inhibitors with anti-PDFGR activity, potentiates the efficacy of NSCLC radiotherapy *in vitro*.

Our findings suggest that radiation survived cells have a complex phenotype combining the properties of CSCs and EMT. CD44, SNAIL and PDGFR-beta are dramatically upregulated in radiation survived cells and might be considered as markers of radiotherapy response in NSCLC.

## Background

Worldwide, lung cancer ranks highest in terms of both incidence and mortality [1]. Despite decades of research, systemic therapies fail to cure most lung cancers. Lung cancers are comprised of two major histological types: small-cell lung cancer (SCLC) and non-small-cell lung cancer (NSCLC, i.e., adenocarcinoma, squamous cell carcinoma, and large cell carcinoma). NSCLC comprises 85% of lung cancer cases and about 40% are unresectable [2].

Radiotherapy used for patients with unresectable NSCLC tumors remains largely palliative due to radioresistance [1,3] which is possibly due to tumor heterogeneity in terms of cell of origin, pathology, etiology and molecular/genetic pathogenesis [4]. The existence of cancer stem/progenitor cells (CSCs) or tumor-initiating cells (TICs) reflects the cellular heterogeneity within solid tumors [5-7]. These cells are the undifferentiated cells with a high tumorigenic and self-renewal capacity, which have been identified in various human malignancies including breast, brain, prostate, pancreatic, colon and lung cancer [8-13]. CSCs express specific markers and stem cell genes and use common signaling pathways including Wnt/β-catenin, Hedgehog and Notch [5-7]. CSCs can be enriched as a subpopulation of cells propagating as non-adherent spheres in medium suitable for tumor stem cells [9]. Evidence suggests that the failure of the treatment may be due to the existence of CSCs which are resistant to chemo- and radiotherapy [14-16].

The cell surface markers CD133, CD44, CD166, as well as ABCG2 transporter and aldehyde dehydrogenase 1 family, member A1 (ALDHA1) protein, have also been reported as human lung CSC markers [12,13,17-20]. Hoechst 33342 dye excluding cells, termed as side-population (SP) cells, has been described as CSCs in a variety of tumor types, including NSCLCs [21,22]. Oct-4 protein is also critically involved in the self renewal of undifferentiated embryonic stem cells and is frequently used as a marker for undifferentiated cells. We demonstrated that lung cancer stem cells that survived treatment with chemotherapeutic drugs express Oct-4 [13]. The role of these important putative stem traits in lung CSCs were confirmed in further studies [23-25]. The expression of CSC-related markers, after chemo and radiation therapy, significantly correlated with a poor prognosis in patients with NSCLC [26], however direct confirmation of the role of lung CSCs in NSCLC radioresistance is missing.

In cancers, epithelial-mesenchymal transition (EMT) is also associated with resistance to chemotherapeutic drugs and radiation [27,28]; epithelial tumor cells with ongoing EMT develop CSC traits [29,30]. EMT is an embryonic process leading to the loss of cell-cell contact, repression of E-cadherin expression and increased cell motility. EMT transition in epithelial cells leads to switching from E- cadherin to N- cadherin [31].

As epithelial cancer cells undergo EMT, they gain stemness, motility, invasiveness, drug resistance and angiogenic and metastatic ability. EMT is regulated by a network of transcription factors including Snail, Slug, Twist, Zeb, and others [29,30,32,33]. EMT can be activated by TGF-β and other oncogenic pathways (e.g., Src, Ras, Notch) [32,34] and by tumor microenvironment stresses (e.g., hypoxia) [35,36]. IR promotes EMT in tumor cell lines via the activation of TFG β signaling [37-40]. The alteration of the expression of the E -cadherin/β-catenin complex is an independent poor prognostic factor in human lung adenocarcinoma [41]. However, until now there have not been any studies that have elucidated the CSC and EMT characteristics of NSCLC cells that survived IR-treatment.

In the present study, we performed an extensive comparative phenotypic analysis of CSC and EMT marker expression in bulk naïve adherent NSCLC cells, in irradiation survived adherent cells, non-irradiated lung tumor sphere cells and radiation–survived NSCLC cells growing as tumor spheres using the High Content Screening Approach (ThermoFisher, Cellomics INC).

Our findings demonstrate the role of CSC and EMT in radiation survived tumor sphere cells and may lead to the identification of the new therapeutic targets.

## Materials and methods

### Cell lines

The human A549 and H460 NSCLC cell lines were purchased from the American Type Culture Collection (ATCC). Cells were grown in culture media, as recommended by the ATCC, that was supplemented with 10% FBS (Millipore Inc., Billerica, MA).

### Reagents

Hoechst 33342 was purchased from Sigma-Aldrich (Sigma-Aldrich, St. Louis, MO). Fluorochrome-conjugated antibody against human CD44 was from Beckman Coulter (Fullerton, CA). Antibodies against PDGFR-a; PDGFR-beta, CD166 and Sox-2 were from R&D Systems INC (Minneapolis, MN). Antibodies against CD24, Snail1, Twist1, Slug, N-cadherin, Vimentin, Fibronectin, ALDH1A1 and pancytokeratin were from Abcam Inc. (Abcam, Cambridge, MA). Oct-4 and E-Cadherin antibodies were purchased from Cell Signaling Technology Inc., (Cell Signaling, Danvers, MA). CD133 antibody was obtained from Miltenyi Biotec (Germany). Alexa Fluor®-488 conjugated mouse antibody against human ß-catenin were purchased from BD Biosciences Inc. (San Diego, CA). Secondary antibody conjugated with Alexa**®-**488, -546, and-680 was from Molecular Probes (Invitrogen, Carlsbad, CA).

The Tyrosine kinase inhibitors, Axitinib and Dasatinib, were purchased from LC Laboratories (Woburn, MA).

### Irradiation

NSCLC cells were irradiated, as cell suspension or as monolayer, using the Shepherd Mark 1 68 Irradiator, (137Cs Irradiator) (JL Shepherd, San Fernando, CA, USA) dose rate of 70.6 rad/min at room temperature.

### In vitro clonogenic assays

Exponentially growing H460 and A549 cells were harvested by exposure to trypsin and cell suspensions were irradiated using a ^137^Cs gamma-ray source with doses ranging from 0 to 10 Gy and plated in 6 well plates, 500 cells per well. Cultures were incubated at 37°C in 5% CO_2_. The next day, axitinib or dasatinib was added to the cultures at a final concentration of 1 μM. The culture media with inhibitors was changed every day. Seven days later the cells were fixed and stained with crystal violet; colonies ≥50 cells were counted and the size of the colonies were measured (Gel Count colony counter, Oxford Optronix, Oxford, UK). Data was analyzed with linear quadratic and single-hit multi-target models [42].

### Culture of irradiated cells

Cells were seeded at a density of 2 × 10^5^ cells/well, in 12-well plates containing RPMI, supplemented with 10% FBS. After overnight incubation, cells were irradiated (0–10 Gy) and maintained at 37°C in the incubator with 5% CO_2_. Medium replacement was done every third day with fresh medium.

To obtain adherent radiation survived cells, cells were kept in the plates for two weeks. To analyze EMT and CSC markers in adherent radiation survived cells, cells were harvested, counted and seeded into 96 well plates containing RPMI, supplemented with 10% FBS. The next day, the cells were fixed, immunofluorescently stained and analyzed as described below. To analyze cell motility, adherent radiation survived cells were collected and seeded into 6 well plates containing RPMI, supplemented with 10% FBS and a wound healing assay was applied.

To generate radiation survived tumor sphere cells, seven days after IR treatment the cells were harvested, filtered, counted and used for lung tumor sphere assays.

### Culture of lung tumor spheres

Suspension growth was assessed in methyl cellulose-based (0.8% MC) medium as described [9,11]. Briefly, NSCLC cells and IR-survived cells were resuspended in 0.8% MC-based serum free medium (Stem Cell Technologies, Vancouver, Canada) and supplemented with 20 ng/mL EGF (BD Biosciences), bFGF and 4 μg/mL insulin (Sigma) and plated at 500-10000cells/mL in ultra low attachment 24–96 well plates (Corning, Corning, NY) for 12 days. The medium was replaced or supplemented with fresh growth factors (EGF, bFGF (20 ng/mL) and insulin (4 μg/mL)) twice a week. The tumor sphere growth was analyzed under a phase-contrast microscope with an ×10 objective and counted from multiple wells.

### Self renewal

In order to assess the self renewing potential of the cells, spheres of the first generation were collected by gentle centrifugation, dissociated into single cell suspensions, filtered through a 30 μm filter and cultured in ultra low attachment plates in stem cell selective medium as described above. The tumor sphere growth was analyzed under a phase-contrast microscope with a 10× objective and counted from multiple wells. To generate spheres of the 3rd, 4th and 5th generations, the 2nd, 3rd and 4th generation tumor spheres were collected; the single cell suspensions were prepared and were replated. The tumor spheres were counted after 12 days.

### Differentiation

Cells dissociated from spheres (third generation) were plated at 1 × 10^4^ cells/mL on 96-well plates that were precoated with Collagen IV (BD Biosciences), in culture media supplemented with 10% FBS and transferred into new plates when cultures reached confluence. To test the self renewing potential of differentiated cells, they were transferred into semisolid serum-free media supplemented with EGF, FGF and insulin and their ability to form tumor spheres was evaluated, as described above. To perform a phenotypic characterization of the cells, from spheres and after differentiation, the cells were seeded in 96 well plates (5 × 10^3^ cells/well) and stained with various antibodies as described above.

### Monolayer wound healing assay

Cells were seeded into the six-well plates at high density and were cultured for 2 days to produce a monolayer. Cells were plated, scratched and monitored in the media supplemented with 10% FBS. Confluent cell monolayers were then scratched using a plastic 10 μL-pipette tip as described [43]. Wounded monolayers were then washed four times with medium to remove cell debris and incubated in a culture medium supplemented with 10% FBS for 24 h. Images were captured in 0, 2, 4, 6 and 24 h using a ZEISS (light) microscope Axiovert 40C (Hamamatsu).

### Cell staining procedure for cellomics arrayscan automated imaging

Cells were fluorescently stained as previously described [13,44]. Briefly, cells grown in 96-well plates were fixed, washed with FACS buffer, incubated with antibodies against CD24, CD34, CD44, CD117, CD166, conjugated with FITC, PE or PC5 for 1 h; fixed in 2% PFA for 20 min, washed in PBS, and stained with Hoechst 33342. To test CD133, PDGFRα, PDGFRβ and CXCR4 expression, cells were incubated with respective primary antibodies and then with secondary antibodies that were conjugated with Alexa 488, 546, or 680 fluorochromes (Molecular Probes/Invitrogen) for 1 h. Cell nuclei were then stained with Hoechst 33342 at 2 μg/ml for 20 minutes to identify individual cells and to optimize focusing. To detect intracellular proteins, cells were fixed and permeabilazed and then stained for ß-catenin, Sox-2, Snail1, Twist1 and cytokeratins using primary and secondary antibodies conjugated with Alexa**®-** 488, 546 or 680 dyes (Molecular Probes/Invitrogen) as described above. All incubation and fixation procedures were performed at room temperature.

### Cellomics array scan automated imaging

The Cellomics ArrayScan HCS Reader (Cellomics/ ThermoFisher, Pittsburgh, PA) was utilized to collect information on the distribution of fluorescently labeled components in stained cells. The ArrayScan HCS system scans multiple fields in individual wells, acquiring and analyzing each of the cell images according to defined algorithms. The scanner is equipped with emission and excitation filters (XF93, Omega Optical, Brattleboro, VT, USA) for selectively imaging fluorescent signals. Data was captured, extracted and analyzed with ArrayScan II Data Acquisition and Data Viewer version 3.0 (Cellomics), Quattro Pro version 10.0.0 (Corel, Ottawa, Ontario, Canada) and MS Excel 2002 (Microsoft, Redmond, WA).

### Statistical analysis

Experiments were performed at least three times. Comparisons between values were performed using a two-tailed Student’s t-test. For the comparison of multiple groups, a one- or two-way ANOVA test was applied. For all statistical analyses, the level of significance was set at a probability of 0.05.

## Results

### Generation of NSCLC cells survived ionizing radiation and propagated as tumor spheres

**Figure 1:**
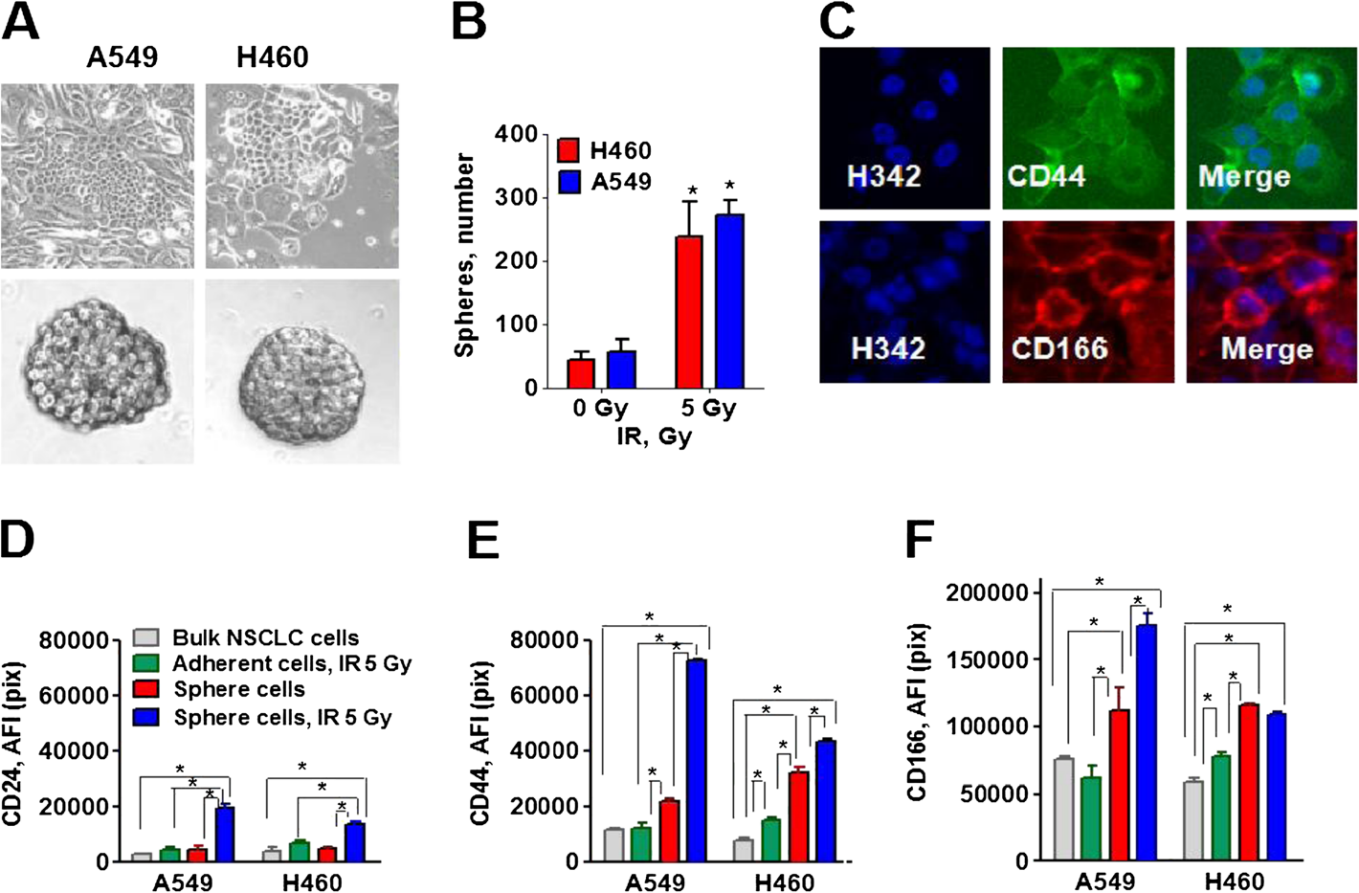
**The radiation survived NSCLC cells express cancer stem cell markers.** A549 and H460 cells were irradiated (5Gy) and cultured in adherent conditions for a week. Cells were collected and plated as single cell suspensions, in stem-cell selective conditions, to form floating tumor spheres. To obtain adherent radiation survived cells, cells were kept in the plates for two weeks after IR-treatment. Expressions of markers were tested in non-irradiated cells, adherent cells that survived IR treatment, non-irradiated sphere cells, and sphere cells that survived irradiation. **(A)** Morphology of adherent cells that survived IR treatment and spheres developed from IR-treated cells are shown. **(B)** Radiation survived cell populations are enriched in the tumor-sphere forming cells. Non-irradiated cells and radiation survived cells were harvested, and transferred into the stem cell selective conditions. 10 days later the tumor spheres, from the first generation, were counted under the microscope. The tumor spheres, from the first generation, were used to develop the tumor spheres for the second generation. The second generations of tumor spheres were used to develop the third generation spheres used for further study. **(C-F)** Analysis of CD44, CD24 and CD166 expression. Cells in 96-well plates were fixed, incubated with the respective antibody and stained with Hoechst33342. Cell images were acquired using the ArrayScan HCS Reader (40× objective) and analyzed using the Target Activation BioApplication Software Module. **(C)** The representative images of the A549 radiation survived sphere cells stained for CD44 and CD166 are shown. **(D-F)** The total average fluorescence intensities of CD24 **(D)**, CD44 **(E)** and CD166 **(F)** in the non-irradiated cells (grey), IR- survived adherent cells (green), non-irradiated sphere cells (red) and in the IR-survived sphere cells (blue) are presented. The fluorescence intensities of respective IgG controls were subtracted. Each point presents average intensities (pixels) estimated for 3000 cells.

A549 and H460 bulk NSCLC cells were treated with clinically relevant doses of IR (5.0 Gy) and cultured for one week. The majority of cells died by the fifth day and enlarged cells were seen with flattened, senescent-like morphology. Radiation survived cells started clonogenic growth (Figure 1A, top images).

On the seventh day after irradiation, the cells were collected, filtered and plated as single cell suspensions in stem-cell selective conditions: ultra-low attachment plates, serum free media supplemented with growth factors to form floating tumor spheres. As shown in Figure 1A, IR-survived A549 and H460 cells proliferated and generated floating clones or tumor spheres.

Growth as tumor spheres is considered to be a surrogate marker for stemness and self renewal ability in epithelial cancers [9]. The ability to form tumor spheres in low attachment growth conditions is a good index of the tumor-forming potential of stem cells. We have analyzed the tumor sphere formation in the radiation survived adherent clones as well as the parental NSCLC cells. As shown in Figure 1B, the numbers of first generation tumor spheres which developed from adherent radiation survived cells, was significantly higher than the number of spheres which developed from non-irradiated parental NSCLC cells.

Next, we developed second and third generation tumor spheres using our published approach [13]. The third generation lung tumor spheres cells were used for further study.

In addition, A549 and H460 cells that survived IR treatment (5.0 Gy), in adherent monolayer cultures, were used in the comparative study.

### Analysis of lung cancer stem cell traits

To determine whether the radiation survived cells have other intrinsic properties of stem cells, immunofluorescent staining and HCA and HCS analyses from Cellomics, ThermoFisher, for expression of CSC and embryonic transcription factors were used. The comparative analyses of CSC markers in non-irradiated NSCLC cells, radiation survived adherent cells, non-irradiated lung tumor sphere cells, and radiation survived cells growing in tumor spheres was performed (Figure 1C-F). Non-irradiated lung sphere cells, as well as radiation survived sphere cells, showed significant upregulation of CD44 and CD166, as compared with parental NSCLC cells in both cell lines, which corresponds to the previously identified phenotypic markers of human lung CSCs. CD44 expression was significantly higher in the radiation survived sphere cells than in non-irradiated sphere cells in both cell lines, whereas CD166 was significantly upregulated in sphere cells generated from IR- treated A549 cells. CD44 and CD166 levels were also higher in radiation survived adherent H460 cells, than in non-irradiated bulk H460 cells.

Interestingly, CD24 upregulation was observed only in radiation survived sphere cells and was not elevated in non-irradiated lung spheres cells or radiation survived adherent cells in comparison to bulk NSCLC cells in both cell lines (Figure 1D).

**Figure 2:**
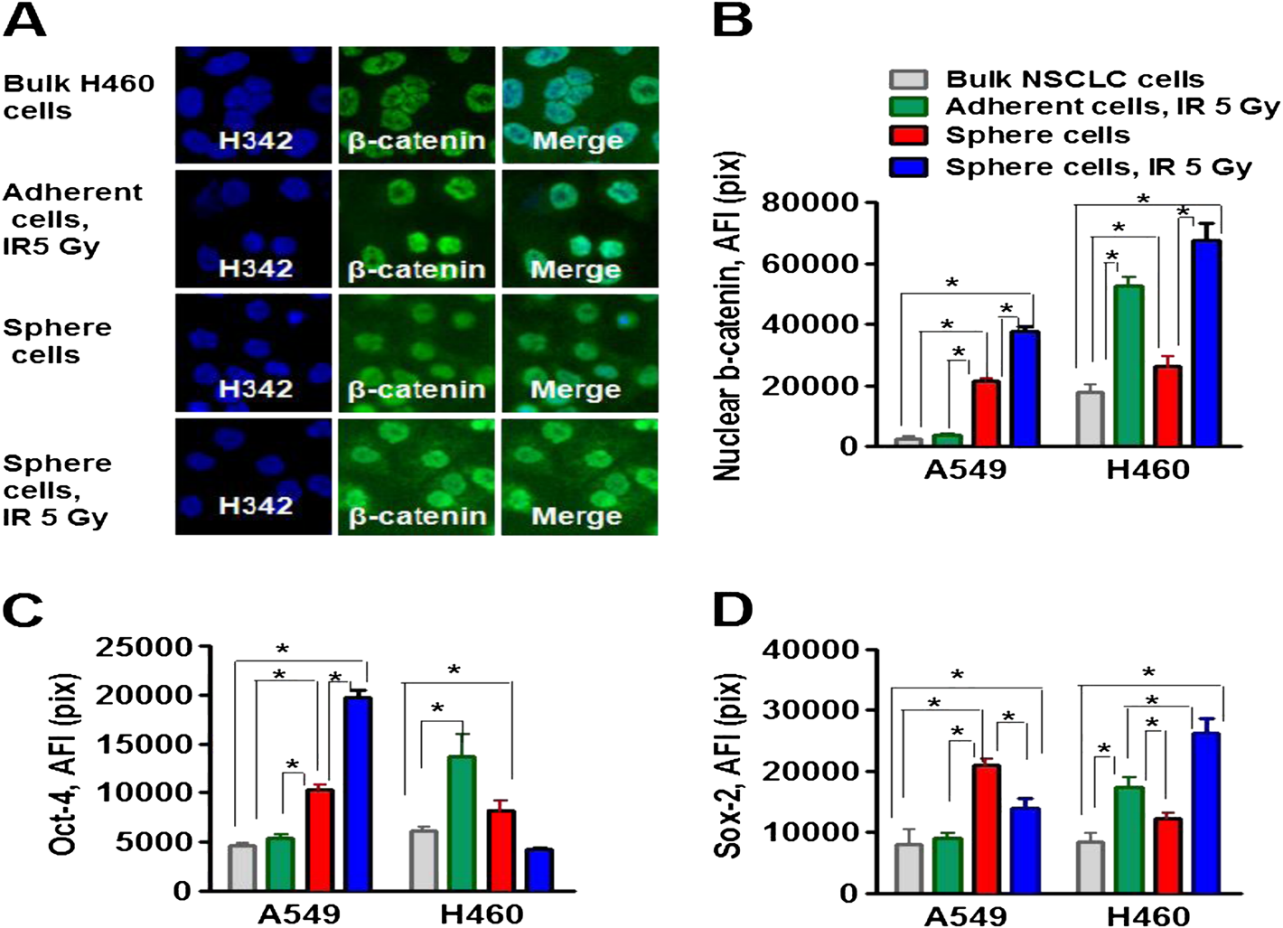
**The radiation survived NSCLC cells show upregulation of beta-catenin, Oct-4 and Sox-2 transcription factors.** Non-irradiated NSCLC cells, radiation survived adherent cells, non-irradiated sphere cells and radiation survived sphere cells were collected and seeded into collagen precoated 96-well plates. 8 hours later the cells were fixed, permeabelized and immunofluorescently stained for beta-catenin, Oct-4 and Sox-2. The cell nuclei were stained with Hoechst33342. Cell images were acquired using the Cellomics ArrayScan HCS Reader (40× objective) and analyzed using the Compartment Analysis BioApplication Software Module. **(A)** The representative images of parental non-irradiated H460 cells, radiation survived adherent cells, non-irradiated sphere cells and radiation survived sphere cells stained for beta-catenin are shown. **(B-D)** The total average fluorescence intensities of nuclear beta-catenin **(B)**, Oct-4 **(C)** and Sox-2 **(D)** in the non-irradiated NSCLC cells (grey), in the radiation survived adherent cells (green), in the non-irradiated sphere cells (red) and in the radiation survived sphere cells (blue) are presented. Fluorescence intensities of respective IgG controls were subtracted. Each point presents average intensities (pixels) estimated for 3000 cells.

We next analyzed the expression of transcription factors correlating with stemness (beta- catenin, Oct-4, and Sox-2) in non-irradiated NSCLC cells, radiation survived adherent cells, non-irradiated sphere cells and in the radiation survived sphere cells (Figure 2). As shown for beta-catenin (Figure 2A), these transcription factors are mainly located in the nuclei of the cells. Beta-catenin upregulation has been detected in the radiation survived sphere cells in both cell lines (Figure 2B). A substantial increase in Oct-4 has been found only in A549 radiation survived sphere cells, whereas upregulation of the Sox-2 transcription factor has been observed only in H460 radiation survived sphere cells (Figure 2C-D). However, in general, the elevated expression of two stemness-associated transcription factors (e.g. β-catenin and Oct-4; β-catenin and Sox-2) in radiation survived sphere cells suggests that these cells might have more pluripotent and aggressive phenotypes than non-irradiated lung tumor sphere cells. Radiation survived adherent H460 cells display upregulation of stemness-associated transcription factors, Oct-4, Sox2 and beta –catenin, whereas radiation survived adherent A549 cells have shown the same low level of these transcription factors as adherent non-irradiated bulk A549 cells.

Radiation survived sphere cells could be propagated for at least 10 passages after mechanical disaggregation of the spheres into a single cell suspension and replating into low-adherence conditions in serum-free media supplemented with growth factors. Limiting dilution experiments showed that the percentage of clonogenic cells, in spheres generated from IR –escapees, was 22% and 27% in cells derived from H460 and A549 cell lines, respectively, and did not change in subsequent passages.

If radiation- survived sphere cells were disaggregated and plated in collagen precoated plates with culture media supplemented 10% FBS, they adhered and started proliferating. After four weeks of culture under adherent conditions in culture media supplemented with FBS, the cells differentiated and acquired an epithelial morphology with decreased expression of CD44, CD24 and CD166 (data not shown).

### Analysis of epithelial to mesenchymal transition (EMT) markers

We studied a number of markers associated with EMT using immunofluorescent staining and the Compartment Analysis BioApplication Software Module from Cellomics which allows us to quantify fluorescence signals in nuclear and cytoplasm compartments of the cells.

**Figure 3:**
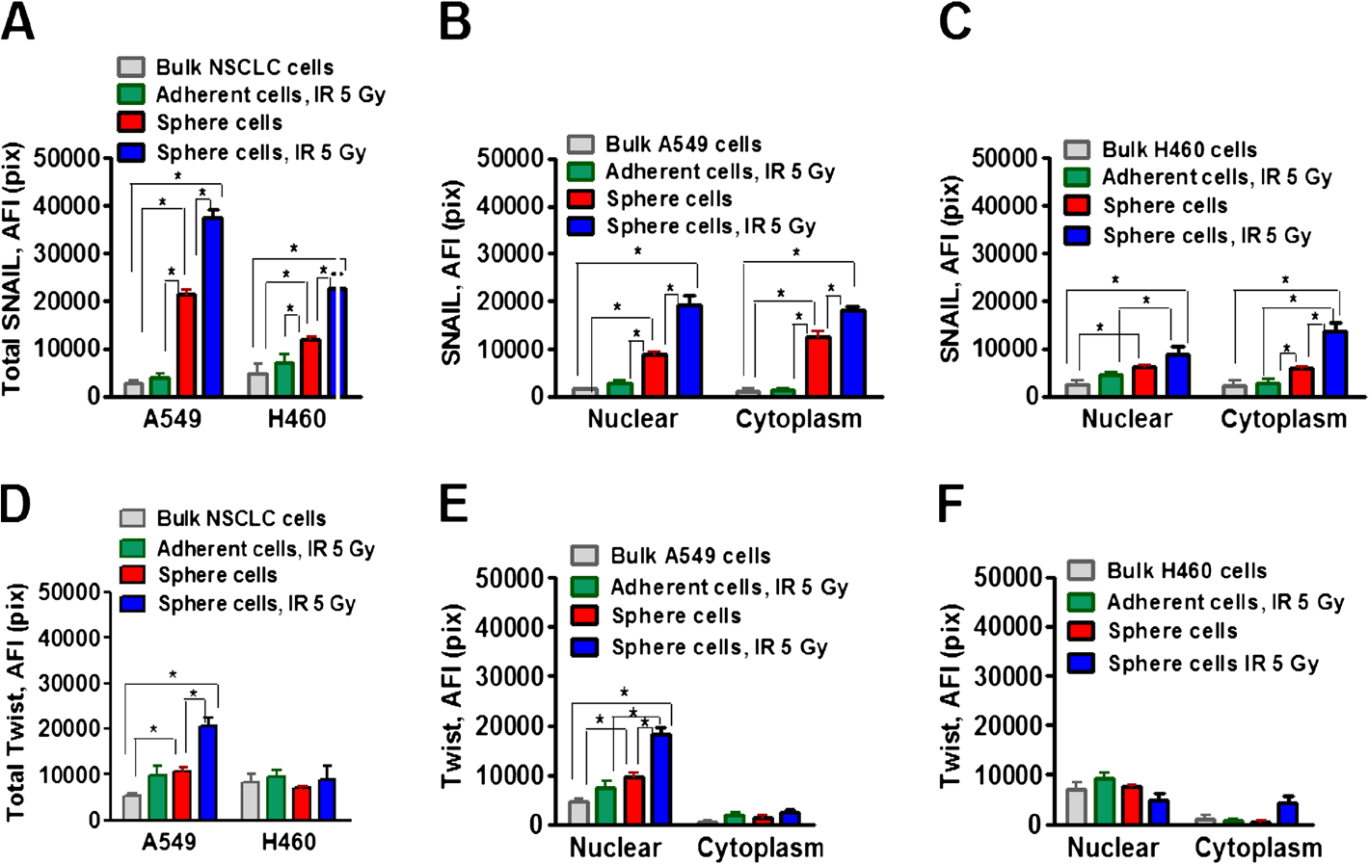
**Upregulation of EMT-associated transcription factors Snail1 and Twist in radiation survived sphere cells.** Non-irradiated NSCLC cells, radiation survived adherent cells, non-irradiated sphere cells and radiation survived sphere cells were collected and seeded into collagen precoated 96-well plates. 8 hours later, the cells were fixed, permeabilazed and immunofluorescently stained for Snail1 and Twist. The cell nuclei were stained with Hoechst33342. Cell images were acquired using the Cellomics ArrayScan HCS Reader (40× objective) and analyzed using the Compartment Analysis BioApplication Software Module. Fluorescence intensities of respective IgG controls were subtracted. Each point presents average intensities (pixels) estimated for 3000 cells. **(A-C)***Analysis of Snail1 expression.* The total average fluorescence intensities of Snail1 **(A)** in the non-irradiated NSCLC cells (grey), in the radiation survived adherent cells (green), in the non-irradiated sphere cells (red) and in the radiation survived sphere cells (blue) are presented. **(B,C)** Snail1 distributions, in the nuclei and cytoplasm compartments of the same cell populations, are shown. **(D-F)***Analysis of Twist expression.* The total average fluorescence intensities of Twist **(D)** in the non-irradiated NSCLC cells (grey), in the radiation survived adherent cells (green), in the non-irradiated sphere cells (red) and in the radiation survived sphere cells (blue) are presented. **(E,F)** Twist distributions, in the nuclei and cytoplasm compartments of the same cell populations, are shown.

Non-irradiated sphere cells, as well as radiation survived sphere cells, showed an upregulation of EMT-related transcription factor Snail1 as compared to adherent parental and IR treated H460 and A549 cells. However, the level of Snail1 expression was significantly higher in radiation survived sphere cells than in non-irradiated sphere cells (Figure 3A). Snail1 was detected in the nuclear and in the cytoplasm compartments of the cells (Figure 3B,C). In contrast, the up-regulation of the EMT-related transcription factor Twist was observed only in the radiation survived sphere cells originated from A549 cell line and Twist was mainly located in the cell nucleus (Figure 3D,E). Radiation survived sphere cells and non-irradiated sphere cells that originated from H460 cell line expressed the same low levels of Twist as the parental H460 cells (Figure 3D,F).

**Figure 4:**
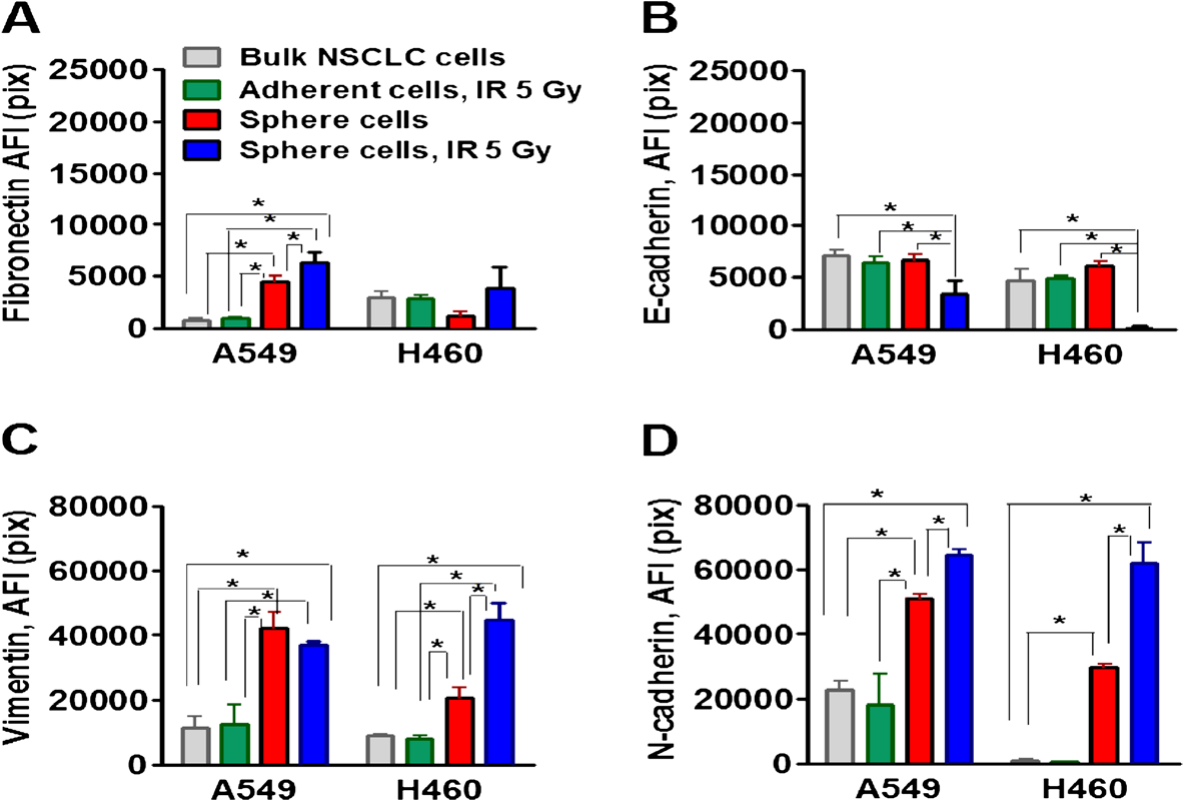
**The radiation survived lung tumor sphere cells show upregulation of EMT markers.** Non-irradiated A549 and H460 cells, radiation survived adherent cells, non-irradiated sphere cells and radiation survived sphere cells were collected and seeded into collagen precoated 96-well plates. Eight hours later, cells were stained for fibronectin, vimentin, N-cadherin and E-cadherin. The cell nuclei were stained with Hoechst33342. Cell images were acquired using the Cellomics ArrayScan HCS Reader (40X objective) and analyzed using the Target Activation BioApplication Software Module. The total average fluorescence intensities of fibronectin **(A)**, E-cadherin **(B)**, vimentin **(C)**, and N-cadherin **(D)**, in the non-irradiated bulk NSCLC cells (grey), in the radiation survived adherent cells (green), in the non-irradiated sphere cells (red) and in the radiation survived sphere cells (blue) are presented. Fluorescence intensities of the respective IgG controls were subtracted. Each point presents average intensities (pixels) estimated for 3000 cells.

To further confirm the EMT phenotype of radiation survived sphere cells, we analyzed the expression of fibronectin, vimentin, N-cadherin, and E-cadherin Figure 4). As shown in Figure 4, non-irradiated sphere cells and radiation survived sphere cells demonstrated strong upregulation of vimentin and N-cadherin when compared with the adherent bulk and IR treated cell populations, however, this EMT marker expression was significantly higher in radiation survived sphere cells than in non-irradiated sphere cells in both cell lines.

Fibronectin was elevated only in sphere cells and radiation survived sphere cells of the A459 cell line but not of the H460 cell line. In contrast, repression of E-cadherin expression was observed in radiation survived sphere cells when compared with bulk NSCLC cells and non-irradiated sphere cells (Figure 4) in A459 and also H460 cell lines.

### Analysis of cell migration

**Figure 5:**
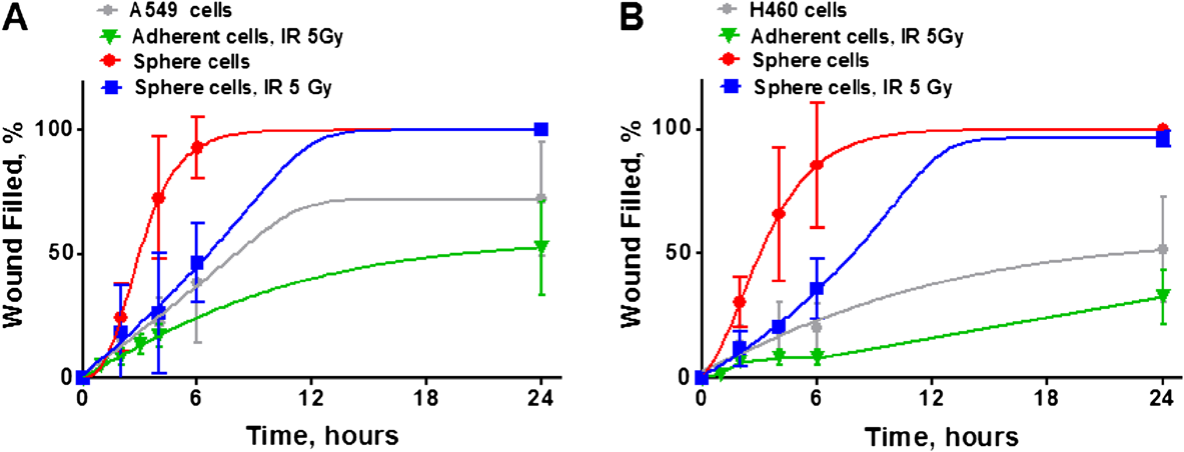
**Wound healing assay demonstrates high migratory potential of the radiation survived sphere cells.** Non-irradiated NSCLC cells, radiation survived adherent cells, non-irradiated sphere cells and radiation survived sphere cells were collected and grown to monolayers in 6-well plates. Then cells were scratched and incubated for 0–24 hours. **(A,B)** The migratory rates of the bulk NSCLC cells (grey), of the radiation survived adherent cells (green), of the non-irradiated sphere cells (red) and of the radiation survived sphere cells (blue) in a wounding assay, were determined by measuring wound width as a function of time. Data are expressed as the mean ± SD of three experiments.

Next, we tested whether EMT marker expression, in radiation survived sphere cells, was associated with increased cell motility. Migratory rates of non-irradiated NSCLC cells, radiation survived adherent cells, non-irradiated lung tumor sphere cells and radiation–survived cells growing in tumor spheres were monitored in an in vitro wound healing assay. As shown in Figure 5, sphere cells, non-irradiated and radiation survived, were able to reestablish a monolayer significantly faster than non-irradiated and radiation survived adherent H460 and A549 cells. For sphere cells, non-irradiated and radiation survived, wounds closure was complete at 24 h after the scratching, whereas adherent NSCLC cells did not complete wound healing at 24 hours. This data indicates that tumor spheres cells have a higher motility than adherent NSCLC cells.

### Analysis of CXCR4 and PDGFR receptors expression

**Figure 6:**
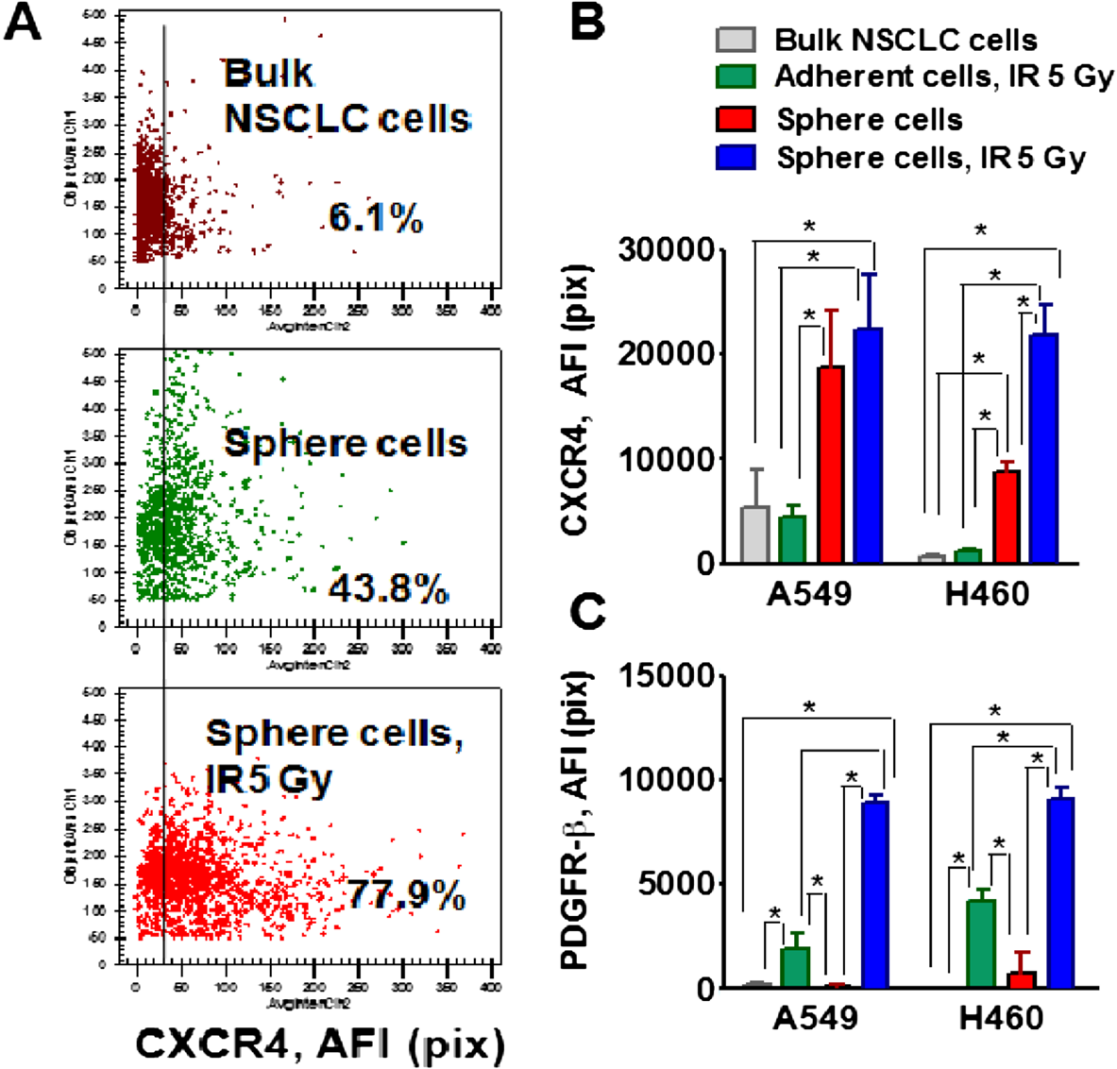
**Radiation- survived sphere cells display upregulation of CXCR4 and PDGFR-beta receptors.** Bulk NSCLC cells, radiation survived adherent cells, non-irradiated sphere cells from the third generation, and radiation survived sphere cells were seeded into collagen precoated 96-well plates for 8 hours. The cells were fixed and immunofluorescently stained for CXCR4 and PDGFR-beta. The cell nuclei were stained with Hoechst33342. Cell images were acquired using the Cellomics ArrayScan HCS Reader (40× objective) and analyzed using the Target Activation BioApplication Software Module. **(A)** Fluorescence intensity of CXCR4 in non-irradiated A549 cells (brown), non-irradiated sphere cells (green), and radiation survived sphere cells (red) plotted against the object area. Each spot represents a single cell**.** Cells to the right of the black line are positive (above IgG control staining). **(B,C)** The total average fluorescence intensities of CXCR4 **(B)** and PDGFR-beta **(C)**, in non-irradiated bulk NSCLC cells (grey), in the radiation survived adherent cells (green), in the non-irradiated sphere cells (red) and in the radiation survived sphere cells (blue) are shown. Fluorescence intensities of respective IgG controls were subtracted. Each point presents average intensities (pixels) estimated for 3000 cells.

Upregulation of CXCR4 is functionally crucial for the maintenance of stemness in drug-resistant NSCLC cells [13,45]. Platelet-derived growth factor receptor (PDGFR) signaling plays a crucial role in specifying the mesenchymal stem cell (MSC) commitment to mesenchymal lineages [46]. Therefore, we investigated CXCR4, PDGFRα and PDGFRβ expression in non-irradiated bulk NSCLC cells, radiation survived adherent cells, non-irradiated sphere cells and radiation survived sphere cells (Figure 6). An increase of expression of CXCR4, in non-irradiated sphere cells and radiation survived sphere cells, was observed (Figure 6A,B). PDGFRα was not detectable in any of the four cell populations investigated (data not shown). PDGFR beta was also undetectable in non-irradiated H460 and A549 cells and non-irradiated lung tumor sphere cells. In contrast, radiation survived cells, adherent cells and sphere cells, in both cell lines showed a significant increase of PDGFR beta expression (Figure 6C).

### The effect of PDGFR inhibition

**Figure 7:**
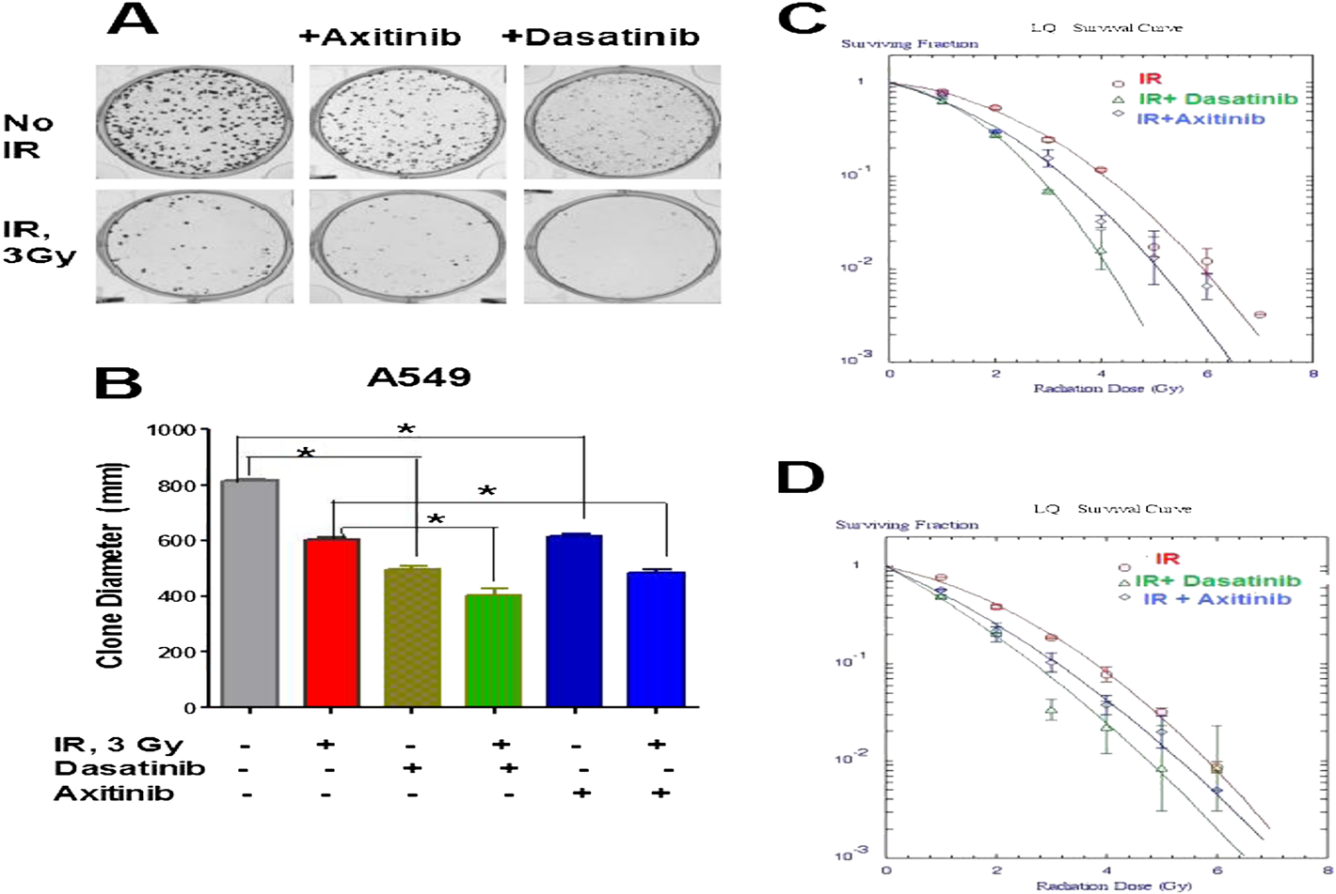
**Clonogenic analysis of cells treated with PDGFR inhibitors and IR.** Cells were suspended, counted, irradiated (0–10 Gy) and plated. The next day, axitinib or dasatinib were added or not into the culture media. Then culture media with inhibitors was changed every day. Cells were fixed and clonogenic survival was estimated on the seventh day after IR treatment. **(A) **Representative images of A549 cell clones generated from IR (3Gy) treated or non-treated cells and growing in presence/absence of axitinib or dasatinib. **(B)** Comparison of clone sizes grown from A549 cells irradiated (3 Gy) or non-irradiated and growing in presence/absence of axitinib or dasatinib. **(C,D)** Radiation survival curves show radiation sensitivity of A549 **(C)** and H460 **(D)** cells growing without inhibitors (red line), growing in the presence of axitinib (1 μM, blue line) or dasatinib (1 μM, green line). Average data of 5 experiments are presented.

To test whether PDGFR beta inhibition will potentiate the effect of IR treatment on NSCLC cells we used axitinib and dasatinib, the tyrosine kinase inhibitors with anti-PDGFR beta activity, in clonogenic survival assay. The results of the clonogenic survival assay presented in Figure 7 and Table 1 demonstrate that combining IR treatment with PDGFR inhibition increases the antitumor effect of ionizing radiation. A significant reduction of the proliferating fraction of the NSCLC cells was determined if irradiated cells were grown in the presence of inhibitors. However, the treatment of irradiated cells with dasatinib resulted in the biggest reduction of clonogenic survival in the both cell lines.

**Table 1:** Comparison of radiosensitivity of NSCLC cells treated with RTK inhibitors

**Cell line**	**A549**			**H460**		
**Treatment**	**IR**	**IR + Dasatinib**	**IR + Axitinib**	**IR**	**IR + Dasatinib**	**IR cp Axitinib**
**D**_**0**_	1.058 ± 0.07	0.920 ± 0.05	1.015 ± 0.03	1.077 ± 0.11	0.914 ± 0.11	1.047 ± 0.12
***p*** (vs IR)	-	0.006	0.149	-	0.009	0.406
**ñ**	2.773 ± 0.43	1.464 ± 0.30	1.892 ± 0.22	4.454 ± 1.72	2.206 ± 1.13	2.630 ± 0.61
***p*** (vs IR)	-	<0.0001	0.027	-	0.011	0.029

Results were calculated with linear quadratic and single hit multi-target models and presented as D_0_ –Gy, the dose required to reduce the fraction of survived cells to 37% and ñ (extrapolation number derived by extension of the linear proportion of the radiation survival curve to the ordinate of the graph using a log/linear plot).

## Discussion

In the present study, we demonstrate that NSCLC cells that survived ionizing radiation treatment have a complex phenotype which includes all of the properties of CSCs and markers of EMT. Evidence has recently been accumulating to support the hypothesis that tumors contain a small population of CSCs, also known as tumor initiating cells, which exhibit stem-like cell properties including self-renewal, tumor sphere formation, differentiation and tumor formation in a xenotransplant model [9,47].

CD166/ALCAM is a 100–105 kD type I transmembrane glycoprotein member of the immunoglobulin superfamily of proteins [48], which has also recently been reported as a CSC marker in colorectal and lung cancers [20,49]. Our finding that CD166/ALCAM expression is increased in non-irradiated A549 and H460 lung sphere cells and even more dramatically upregulated in radiation survived sphere cells of the A549 cell line suggests that CD166/ALCAM might serve as a marker for the detection of CSCs and radioresistant counterparts within NSCLC tumors and bears the potential to predict the outcome of radiotherapy by assessment of CSC density. We demonstrate here that the elevated level of CXCR4 expression could also be considered as a marker for non-irradiated human lung sphere cells and also for radiation survived sphere cells. Previously, the upregulation of CXCR4, a receptor of stromal derived factor (SDF/ CXCL12), has been reported as functionally crucial for the maintenance of stemness in drug-resistant NSCLC cells [13,45].

CD44, a receptor for hyaluronic acid and a well known stem cell marker in many epithelial cancers, is a cell-surface glycoprotein involved in cell–cell interactions, cell adhesion and migration [50,51]. CD44 can also interact with other ligands including growth factor receptors such as EGFR2 and PDGFR, osteopontin, collagens and matrix metalloproteinases (MMPs) which are involved in tumor progression and metastasis [52]. CD44 gene transcription is, at least in part, activated by beta-catenin and Wnt signaling [53]. A recent study discovered CD44 as a marker which significantly correlates with response to radiotherapy in early stage larynx cancer patients, both at the mRNA and protein levels [54].

As both CD44 and nuclear beta- catenin are dramatically upregulated in radiation survived lung cells, we hypothesize that CD44/beta-catenin expression might serve as another predictive marker for recurrence after NSCLC radiation therapy.

Oct-4 and Sox-2 are stem cell transcription factors [55]. Our finding that Oct-4 and Sox-2 are highly upregulated in radiation survived sphere cells from the A549 cell line and highly upregulated in radiation surviving adherent H460 cells, in comparison to respective naïve lung adherent NSCLC cells and sphere cells, is suggesting that radiation treatment may select for aggressive tumorigenic phenotype, and that this selection is cell line specific*.* Recently, it was reported that ionizing radiation induced a breast CSC phenotype in non-stem cell populations. This transition was Notch-dependent and coincided with up-regulation of Oct-4 [56].

EMT can be a player in cancer initiation, promoting the clonal expansion of premalignant epithelial cells [57]. Cancer cells undergoing EMT acquire the capacity to migrate, invade the stroma and metastasize. During metastasis, the EMT program enables these cancer cells to propagate from a primary tumor and also supports progression from micro- to macro-metastases [58,59]. We found that radiation survived sphere cells also show upregulation of the signal transducer CD24, whereas non-irradiated sphere cells display very low levels of CD24. It is conceivable that CD24 upregulation is discriminating the EMT phenotype in radiation survived cells. In many tumor types, CD24 expression is associated with metastasis [60]. Recently, CD24+ ovarian cancer cells exhibiting EMT phenotype were reported [61]. EMT is an embryonic process leading to loss of cell-cell contact, repression of E-cadherin expression and increased cell motility. EMT transition in epithelial cells leads to switching from E-cadherin to N-cadherin [31]. In cancers, EMT is also associated with resistance to chemotherapeutic drugs and radiation [27,28], and epithelial tumor cells undergoing EMT may develop CSC traits [29,30].

Our observation is that lung sphere cells, non-irradiated and radiation survived cells, have higher motility in comparison to adherent non-irradiated and radiation survived NSCLC cells, as detected by a wound healing assay *in vitro,* as well as upregulation of EMT associated markers in radiation survived lung sphere cells, which clearly indicates that radiation survived sphere cells have a very complex phenotype combining both human lung CSC and EMT characteristics.

Radiation survived sphere cells demonstrated downregulation of E-cadherin and upregulation of N-cadherin, fibronectin and vimentin in comparison with parental A549 and H460 cells thus confirming EMT activation in the cells. The E-cadherin promoter is repressed directly or indirectly by specific developmental transcription factors such as Twist1 and Snail1, disrupts the polarity of epithelial cells and maintains a mesenchymal phenotype [59,62]. Interestingly, N-cadherin, vimentin and Snail1 upregulation were also observed in the non-irradiated sphere cells, however, the levels of these proteins were significantly higher in the radiation survived sphere cells. Snail1 is a zinc-finger transcription factor belonging to the Snail super family and it is characterized by a strongly conserved carboxy-terminal region containing four to six C2H2-zinc fingers. Snail1 acts as a transcriptional repressor, when the fingers bind to E-box motifs (5′-CANNTG-3′) in target promoters, including the E-cadherin gene (*CDH1*) promoter. Snail is a critical transcriptional repressor of E-cadherin and its expression induces EMT [29,63]. Snail upregulation is associated with radioresistance and chemoresistance in epithelial tumors [64,65]. Twist is a basic helix-loop-helix (bHLH) transcription factor, which plays critical roles throughout development, including influencing mesoderm formation, neurogenesis, myogenesis and neural crest cell migration and differentiation [66]. A number of reports have implicated Twist1 in oncogenesis through its ability to inhibit DNA damage-induced apoptosis and promote metastasis through the induction of EMT [67]. Increased Twist1 expression is correlated with the increased risk of metastasis and poor prognosis in a number of solid tumor types including breast, prostate, ovarian and lung [68,69].

The radiation survived sphere cells originated from both A549 and H460 cell lines expressed a high level of Snail1, whereas Twist upregulation was observed only in radiation survived cells that originated from the A549 cell line. This finding suggests differential levels of EMT activation in radiation survived cells and that the radiation survived sphere cells originating from the A549 cell line have a more advanced EMT phenotype. This suggestion is in concordance with the reports that Snail1 plays an important role in inducing the first EMT steps that lead to the initiation of the invasive process, whereas Twist1 has a critical role in the development of distant metastases by prompting breast cancer cells to enter the bloodstream [63,70]. A combination of EMT and stem cell–like phenotypes is an important predictor of aggressive biologic behavior and has an independent prognostic value in predicting the outcome of primary gastric cancer [71]. However, additional studies are necessary to clarify the impact of these cell subpopulations in NSCLC radiation resistance.

Platelet derived growth factor (PDGF) and its receptors play an important role in tumor cell migration and vasculature formation, and these molecules are also well known drivers of mesenchymal cell proliferation [72,73]. We revealed that PDGFR- beta was undetectable in non treated parental cells and non-irradiated lung sphere cells; however it was highly expressed in radiation survived sphere cells and adherent radiation survived cells. PDFGR- beta is a receptor tyrosine kinase (RTK) whose downstream signaling is effectively abrogated by second generation RTK inhibitors, such as Axitinib (AG-013736) [74] and dasatinib [75,76]. Using a classical clonogenic assay for testing the effect of ionizing radiation, axitinib, dasatinib and the combination of IR with axitinib or dasatinib treatment, we observed that RDGFR signaling may be important for NSCLC radiation resistance as the combination of IR with dasatinib or axitinib treatment significantly increases the efficacy of IR treatment in NSCLC cells. However, combining IR with dasatinib exerts a more profound effect than the combination of IR with axitinib.

In conclusion, the present study demonstrates that NSCLC cells that survived IR treatment and propagated as tumor spheres have a complex phenotype which includes f the properties of CSCs. They have a significant upregulation of CD166, CXCR4, CD44, nuclear beta-catenin, Oct-4, and/or Sox-2 expression and they acquire motility. Radiation survived cells also show an EMT phenotype, including a reduction of E-cadherin and an upregulation of N-cadherin and vimentin. These cells also demonstrate an upregulation of Snail1, a major transcription factor involved in EMT activation and promotion.

PDGFR-beta signaling, which is specifically upregulated in radiation survived sphere cells and IR-treated adherent cells, could be considered as prognostic markers for NSCLC radiation therapy and they may also serve as the targets for improving the efficacy of NSCLC therapy.

Further *in vivo* studies of radiation survived cells may provide key information on relevant pathways to be targeted to increase the radiation response in NSCLC.

## Competing interests

The authors declare that they have no competing interest.

## Authors’ contribution

VL and MG conceived the idea; RG, CB, LB, PB and ME performed the experiments; RG, ME and VL designed the experiments and analyzed the data. VL wrote the manuscript. All authors read and approved the manuscript.
